# Prevalence and risk factors of diarrheal diseases in Sierra Leone, 2019: a cross-sectional study

**DOI:** 10.11604/pamj.2022.41.3.32403

**Published:** 2022-01-03

**Authors:** Doris Bah, Gebrekrstos Gebru, Jean Leonard Hakizimana, Uzoma Ogbonna, Bockarie Sesay, Binta Bah, Paul Mansaray, Joseph Charles, Aiah Jimmy, Amara Leno, Fatmata Jalloh, Nyuma Sengu, Solomon Sogbeh, Hamidu Mansaray, Lansan Kanneh, Adel Hussein Elduma

**Affiliations:** 1 Ministry of Health and Sanitation, Freetown, Sierra Leone,; 2 Sierra Leone Field Epidemiology Training Program, Freetown, Sierra Leone

**Keywords:** Prevalence, diarrheal diseases, households, Sierra Leone

## Abstract

**Introduction:**

many studies have shown that unimproved water sources, inadequate sanitation facilities and poor hygiene are the main causes of diarrheal diseases, especially in developing countries. The aim of this study was to determine the prevalence and risk factors associated with diarrheal diseases in Sierra Leone.

**Methods:**

a cross-sectional study was conducted in March 2019. We used a questionnaire to collect data from study participants. Descriptive statistical analysis was followed to determine frequencies and percentages. Univariate analysis was used to find any association between dependent variable and independent variables. Independent variables that had an association in univariate were included in the multivariate model.

**Results:**

we surveyed 1,002 households (516 in rural and 486 in urban), and 2,311 respondents in four districts. The main source of income was farming 437 (43.6%). A total of 49 (54.2%) households earned below the national minimum wage per month. Females represented 61.9% of respondents. A total of 242 (32.2%) households had one to five household members and 229 (30.5%) households had more than ten members. Around 88.9% of households in urban, and 42.2% rural areas use improved water sources. The prevalence of diarrheal diseases was 12.3%. Multivariate analysis showed that using of unimproved water sources (aOR=1.9; 95% CI, 1.01 to 3.63, p=0.045), and large family size (aOR= 2.5; 95% CI, 1.18 to 5.35, p=0.017) were associated with diarrheal disease.

**Conclusion:**

we concluded that the risk factors associated with diarrheal diseases included unimproved water sources and large family size. More efforts required to improve water resources, adequate sanitation, and hygiene, particularly in rural areas.

## Introduction

Access to safe, adequate and usable drinking water, proper sanitation facilities, and sanitization practices are the foundation of human health and well-being. Safe water, sanitation and personal hygiene are essential to prevent the spread diseases [1]. Inadequate water, sanitation, and hygiene is responsible for 7% of the global burden of disease as measured by disability-adjusted life years (DALYs), and accounts for more than 4% of all deaths worldwide, most of whom are identified as children in developing countries [2].

Various studies have been conducted in sub-Saharan Africa to assess factors influencing water, sanitation, and hygiene (WASH) and transmission of diseases related to water quality and sanitation. sub-Saharan Africa has low water and sanitation coverage, and people living in rural areas are less likely to have access to improved water and sanitation facilities. A study conducted in rural South Africa reported that households (HHs) storing water for human consumption are at high risk of diarrheal diseases [3]. Results showed that the persons interviewed understood the situation and risks that follow inadequate WASH, but largely lacked access to improved WASH [4].

Another study conducted in Nigeria among children under five assessed the impact of household risk factors related to the severity of diarrheal diseases. The incidence of diarrheal disease in rural areas (67.3%) is much higher than that in urban areas (32.7%), which is related to the lack of improved water and sanitation facilities [5]. The 2015 Millennium Development Goal (MDG) report on the improvement of drinking water and sanitation stated that Sierra Leone had achieved good progress to meet the drinking water target, but limited progress for the sanitation target. Roughly, 50-75% of the population uses improved drinking water sources, but less than 50% uses improved sanitation [6].

Globally, unsafe water, inadequate sanitation facilities and poor hygiene practices cause 60% diarrheal diseases. WASH attributed diarrheal disease deaths account for 5% of all deaths among children under five years of age [7]. In Sierra Leone, 66.2% and 73.0% of population living in urban and rural areas use unimproved water sources, respectively. In addition, a high contamination rate have been detected in the drink water sources in households in both urban and rural areas [8]. In Sierra Leone, the mortality rate due to unsafe water, unsafe sanitation and lack of personal hygiene was estimated at 81.3 per 100,000 populations, indicating a high death burden. There is evidence that exposure to unsafe water, and poor hygiene are also main drivers of diarrheal diseases and other intestinal diseases [9].

Despite the huge investments made by the Sierra Leone government, the expected results of improving water sources, sanitation and personal hygiene have not been achieved, which increases the risk of diarrheal diseases. Although several studies have been conducted in sub-Sahara Africa on water, sanitation and personal hygiene and risk factors associated with diarrheal diseases, no studies have been conducted in Sierra Leone. Therefore, this study aims to assess the prevalence of diarrheal diseases at the household level in rural and urban areas of Sierra Leone and to identify risk factors related to diarrheal diseases. The specific objectives included distribution of diarrheal diseases in rural and urban areas. Also, we have determined the association between unimproved water, unimproved sanitation facilities, family members, water sources and the occurrence of diarrheal diseases.

## Methods

**Study design:** a cross-sectional study was conducted to determine risk factors associated with diarrheal diseases in Sierra Leone.

**Study setting and population:** Sierra Leone is located in West Africa, bordering Guinea to the northeast, Liberia to the southeast, and the Atlantic Ocean to the south-west. Sierra Leone is divided into five regions including Eastern, Northern, North West Southern, and Western Area. Twelve of the sixteen districts are divided into 190 chiefdoms and 10,017 enumeration areas (EAs). According to the 2015 Sierra Leone Population and Housing Census, 59% of the total population lives in rural areas compared with 41% in urban areas. The country´s estimated total population was 7,701,185 in 2018, with an area of 71,740 km (27,699 sq. miles) [10]. The study was piloted from March 10 to 12, 2019. Field data was collected from Freetown between 17^th^ to 22 of March 2019, while in Districts data was collection was started on the 23 of March and completed on 31^st^ of March 2018. Most people living in urban areas used chlorinated piped water sources, while household in rural areas drink well water and store water in open container. Households in rural areas do not have improved latrines, as a result they defecate in open place. For those who have toilets in rural areas, they share these facilities with their neighbors. The common method of waste disposal was designated open dumping followed by undesignated open dumping. household had soap at hand washing station and the proportion of households having soap was higher in urban compared to rural area.

The study population were households members. A household was defined as one or more persons living in the same dwelling and sharing meals. For households with less than six members, all members of the household were selected to participate in the study. For households with more than six members, a maximum of six family members was interviewed. Participants this study were included adults (≥18 years), child assent (<18 years) and school chidden (6-17 years of age). All households in the selected areas were eligible to be included in the study. Additionally, household members with more than five years were included in the study.

**Variables:** variables included drinking water, sources, sharing facility, and hand washing. Also, variables included soap provision, history of diarrheal diseases. Unimproved drinking water facility includes non-piped supplies as well as unprotected wells and springs, while “Unimproved sanitation” is comprised of on-site sanitization pit latrines without slabs, hanging latrines, and bucket latrines. Diarrheal infection is the outcome variable, which was defined as having diarrheal diseases in the two weeks prior to household survey. Independent variables included unimproved water resources, improved sanitation, number of household members, household income, and water container. We considered possible confounders in our analysis.

### Data resource and measurement

**Data collection tool:** a structured questionnaire adapted from the water, sanitation, and hygiene knowledge, attitudes and practices (KAP) of the office of the United Nations High Commissioner for Refugees (UNHCR) standard questionnaire [11].

**Data collection:** demographic variables such as sex, age, education, number of person in a household and family income were collected. Also, the data collection tool, collected independent variables such as water sources, sanitation, and water storage. The data was collected through questionnaire, which was pre-tested using sample size of 5% from rural and urban areas. Data was collected by field epidemiology training program students after receiving an intensive training course in data collection. Team members were trained on electronic collection of data on tablets, selection of households, requesting the participation of household members, administration of informed consent and assent, and administration of the questionnaire. Data collection teams collected used structured questionnaire in an electronic (Epi-Info) format to collect data from household members through a face-to-face interview. The data was downloaded from the tablets to the computer. Demographic, clinical, and epidemiological data was downloaded and merged into a database using Epi-Info version 7. The database has been stored in a password-protected computer at the program office. Further data cleaning, as well as analysis, has been done using the same software.

**Sample size:** sample size was calculated using a single proportion, households in urban and rural areas were selected. The proportion of rural respondents using improved sanitation facilities was 27.5% for rural, and the proportion for urban respondents was and 74% [8]. Using design effect 1.5, two-sided confidence limits as (d) =5%, considering 10% of non-response rate, a sample size (n) of 510 for rural, and 494 for urban was calculated. The multistage clustering sampling technique was used to reach the final required sample size. Using systematic sampling technique, the households were selected from the sampling frame of households. First, we selected four districts, one from each region using simple random sampling; Tonkolili, Kenema; Moyamba and Western Area Urban districts. We stratified each selected district into rural and urban. A total of 30 Enumeration Areas (EAs) were selected per district, the number of EAs per stratum was determined according to PPS of EAs in the urban/rural strata in each district. Form our sampling frame, which is a list of all EAs, we selected the EAs using sampling interval. We enrolled a fixed number of households from each EA for the interviews. In the EAs, sampling interval (K) was calculated by dividing the total number of households in the EA by the number of households to be enrolled in that EA. Based on the calculated sample size, 171 households were from Kenema, 184 from Tonkolili, and 155 from Moyamba were selected from the rural stratum. For urban stratum 78 households from Kenema, 30 from Tonkolili, 7 from Moyamba and 377 from Western Area were selected from this stratum.

**Data analysis:** in order to have high quality data, investigators followed a restricted approach during data collection phase. Variables with missing data were discarded from the analysis. Statistics analysis was performed using Epi-Info 7. The dependent variable was the history of diarrheal diseases. The independent variables included demographic, water sanitation and hygiene variables. Descriptive analysis was run, numbers and frequencies were reported. For quantitative variables such as age, we calculate median and range. Analytical statistics was conducted to measure the association between independent and dependent variables using univariate analysis. We followed backward stepwise approach to select variables to build the best model. Frist bivariate analysis was conducted for each independent variable. Assuming there is no interaction, the independent variables with significant association in bivariate analysis (p<0.05), were included in multivariate model. We have conducted removing of independent variables strategy was to select the best model. The best multivariate model was selected and dependent variables with less than p <0.05 were considered as statistically associated with dependent variable. Then, we remove variables that have discarded from the bivariate model, one-by-one check whether any remarkable change occurred in the multivariate model.

**Ethical consideration:** this study was approved by Sierra Leone Research Ethical Committee (Version: 22 March 2019). All adults ≥18 years of age were asked to give informed consent and sign a consent form before participating in the study. For participants who were not able to read, we read and explained the consent for them. For children under five years of age, parents or guardians answered questions about health status and behaviors. For children aged 6-17, everyone was required all were asked to give informed assent and sign an assent form. Children were interviewed in a separate room or area to ensure independent responses. No personally identifiable information was collected and confidentiality was ensured. The electronic data has been stored in a password-protected computer.

## Results

**Socio-demographic analysis:** a total of 1,002 households (516 in rural and 486 in urban) in four districts and 2,311 respondents were interviewed in the surveyed households with median age of respondents 14 years (range: 5 months-96 years). Children of 5 years and below constituted 617 (26.7%), children between 6 to 17 years 686 (29.7%), while adults 18 years and above accounted for 1008 (43.6%). Females represented 1,432 (61.9%) of the respondents. Of the total respondents, 1,124 (48.6%) did not attend school. Muslims represented 1703 (73.7%) of the respondents. The main source of income was farming 437 (43.6%). Four hundred and ninety-seven (54.2%) households earned below the national minimum wage per month. A total of 242 (32.2%) households had one to five household members and 229 (30.5%) had more than 10 members. Further, 550 (54.8%) households had one to three children under 5 years old, while 154 (15.4%) households had more than three children under 5 years.

**Figure 1:**
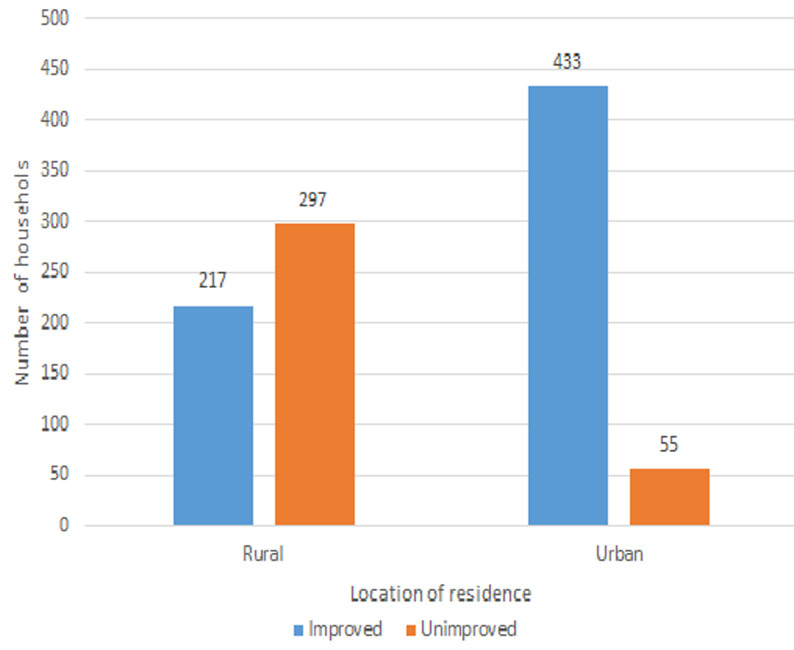
improved versus unimproved water source by location of residence, Sierra Leone, 2019

**Descriptive analysis:** the proportion of households using improved water sources was higher in urban (88.9%) compared to rural setting (42.2%), <0.001. Of 937 (93.5%) respondents fetching water out of their houses, 573 (66.1%) had sources of drinking water at less than 100 meters away from their houses. The percentage of improved water sources in the urban setting was higher (66.6%, n=433) compared to the rural setting (33.4%, n=217) (Figure 1). The survey found that 45(4.5%) households had hand washing devices in their houses. The proportion in urban (93.2%, n=450) compared to rural setting (2.3%, n=12). Only 43.9% households had soap at hand washing stations and the proportion of households having soap was higher in urban. Of households surveyed, 173 (17.3%) had at least one case of diarrheal disease during the 2 weeks prior to household survey administration. Out of the 926 respondents above 18 years, the prevalence was 11.9% (n=110), of 684 respondents of 6 to 17 years, the prevalence was 12.4 %( n=82), and of 617 under five children, the prevalence was 14.9% (n=92), higher in rural (20%) compared to urban area (8.6%). Of 287 respondents who got diarrheal disease, 156 (54.4%) sought medical care, and 270 (94.1%) took medicine.

**Bivariate analysis:** the bivariate analysis of the risk factors associated with diarrheal diseases showed that households in rural settings were 1.6 more likely to have diarrheal disease than those in urban settings (OR=1.6, 95% CI= 1.2-2.3, p=0.003). People washing hands with soap were less likely to get diarrheal disease than those who didn’t use soap (OR= 0.6, 95% CI= 0.3-0.9, p=0.02). People who wash hands after handling child stool (OR: 0.5, 95% CI= 0.3-0.9; p=0.03), and those washing hands before cooking (O.R: 0.6, C.I: 0.4 to 0.8; p=0.0006) were less likely to get diarrheal disease. At individual level, those using unimproved water supplies were more likely to have diarrheal disease than those using improved water sources (OR= 1.6, C.I: 1.2 to 2.1, p<0.001). Households using unimproved water supply were more likely to have diarrheal disease than those using improved water supply (OR=1.9, 95% CI= 1.4-2.7; p= <0.001). At household level, those using unimproved toilet were more likely to get diarrheal disease (OR=2, 95% CI= 1.2-3.5; p=0.007) and households sharing a toilet were more likely to have diarrheal disease (OR=1.5, 95% CI= 1.1-2.3; p=0.03).

Those who reported they treated water were more likely to have diarrheal disease (OR=1.8, 95%CI=1.2-2.8, p=0.005) and those who said they collected enough water were less likely to get diarrheal disease (OR=0.5, 95% CI= 0.3- 0.7, p<0.001). Respondents of 18 years and above with good knowledge on the most important time when people wash their hands were less likely to get diarrheal disease compared to those with poor knowledge (OR=0.6, 95% CI= 0.4-0.9, p=0.027). Respondents with limited hand washing facilities were more likely to get diarrheal disease than those having basic hand washing facilities (OR=2.3, 95% CI= 1.1-4.7, p=0.01) (Table 1, Table 1 (suite), Table 1 (suite 1)).

**Table 1:** bivariate analysis for risk factors in relation to diarrheal diseases, Sierra Leone, 2019

Variables	Diarrheal disease		OR	95% C.I	P-value
	Yes	No			
**Sex**					
Female	71	397	0.8	0.5-1.0	0.1
Male	102	432	Ref		
**Location of residence**					
Rural	106	408	1.6	1.2-2.3	0.003
urban	67	421	Ref		
**Collecting enough water for household**					
Yes	63	439	0.5	0.3-0.7	<0.001
No	104	360	Ref		
**Toilet facility shared**					
Yes	110	511	1.5	1.0-2.3	0.03
No	38	272	Ref		
**Well mouth above the ground level**					
Yes	27	125	0.7	0.4-1.2	0.2
No	45	145	Ref		
**Cover over the well mouth**					
Yes	25	107	0.8	05-1.4	0.5
No	46	162	Ref		
**Can water enter from surface flow**					
Yes	46	155	1.3	0.8-2.3	0.3
No	25	111	Ref		
**Can domestic animal approach the well**					
Yes	36	136	1.1	0.6-1.9	0.7
No	31	129	Ref		

**Table 1(suite):** bivariate analysis for risk factors in relation to diarrheal diseases, Sierra Leone, 2019

Pollution within 10 meters of the well					
Yes	31	134	0.7	0.4-1.2	0.2
No	44	132	Ref		
**Is water chlorinated**					
Yes	32	214	0.9	0.5-1.7	0.9
No	24	154	Ref		
**Water supply interrupted over past year**					
Yes	45	274	1.5	0.8-3.1	0.2
No	11	103	Ref		
**Flooding affect water supply**					
Yes	61	207	2.0	1.3-2.9	<0.001
No	63	423	Ref		
**Water source dry up during dry season**					
Yes	76	288	1.9	1.2-2.8	0.001
No	49	351	Ref		
**Availability of hand washing facility in your house**					
Yes	7	38	0.9	0.4-2	0.7
No	165	785	Ref		
**Availability of hand washing facility at latrine**					
Yes	9	76	0.5	0.2-1.1	0.07
No	163	725	Ref		
**Availability of soap at hand washing station**					
Yes	4	21	0.7	0.2-2.6	0.6
No	7	25	Ref		
**Household income**					
Below the minimum wage (500,000 Le)	91	421	1.2	0.9-1.8	0.2
Minimum wage and above	60	345	Ref		

**Table 1(suite 1):** bivariate analysis for risk factors in relation to diarrheal diseases, Sierra Leone, 2019

Household received health or hygiene messages					
Ever received	145	628	1.6	1.1-2.5	0.02
Never received	28	201	Ref		
**Protect food from flies**					
Yes	106	505	1.0	0.7-1.4	0.99
No	66	314	Ref		
**Source of drinking water**					
Improved	90	560	0.5	0.4-07	0.00001
Unimproved	83	269	Ref		
**Container to collect water**					
Covered	83	489	0.6	0.5-0.9	0.007
Uncovered	90	340	Ref		
**Clean water container**					
Regular	168	783	1.0	0.3-3.5	0.001
Irregular	3	14	Ref		
**Household do anything to treat drinking water**					
Yes	35	101	1.8	1.2-2.7	0.007
No	135	693	Ref		
**How many people in the household**					
≤5 (242)	30	272	Ref		
6-7 (109)	76	93	1.2	0.6-2.3	0.5
8-10 (171)	36	135	1.9	1.1-3.2	0.01
>10 (229)	34	117	2.0	1.2-3.5	0.009
**Highest level of education**					
None	91	400	Ref		
Primary	21	99	0.9	0.6-1.6	0.8
Junior sec	20	99	0.8	0.5-1.5	0.6
Senior secondary and above	41	301	0.8	0.5-1.2	0.3

**Multivariate analysis:** all factors that were significant in bivariate analysis were put in multivariate analysis and only two were found to be statistically associated with the occurrence of diarrheal disease. Households using unimproved water sources were more likely to have diarrheal disease than those using improved water sources (aOR=1.9, 95% CI= 1.02-3.63, p=0.045). High size of the household was also independently associated with the occurrence of diarrheal disease in the household. Households having between eight to ten members are more likely to get diarrheal diseases than those having five member or less (aOR=2.3, 95% CI: 1.10 to 4.92, p value=0.017). Also, the likelihood of having diarrheal was higher in household with more than ten people compared to those with having five members or less (aOR=2.5, 95% CI: 1.10-4.92, p=0.026) (Table 2).

**Table 2:** multivariate logistic regression for risk factors associated to diarrheal disease prevalence, Sierra Leone 2019

Variables	Diarrheal disease		aOR	95% C.I	P-value
**Location of residence**					
Rural	106	408	1.4	0.75-2.85	0.259
urban	67	421	Ref		
**Water source**					
Unimproved source of water	83	269	1.91	1.01-3.63	0.045
Improved source of drinking water	90	560	Ref		
**Collecting enough water for household**					
Yes	63	439	0.6	0.39-1.16	0.154
No	104	360	Ref		
Shared toilet facility					
Yes	110	511	1.2	0.70-2.16	0.461
No	38	272	Ref		
**Flooding affect water supply**					
Yes	61	207	1.2	0.70-2.16	0.461
No	63	423	Ref		
**Water source dry-up during dry season**					
Yes	76	288	1.4	0.81-2.42	0.225
No	49	351	Ref		
**Container to collect water**					
Covered	83	489	1.3	0.73-2.25	0.374
Uncovered	90	340	Ref		
**Clean water container**					
Regular	168	783	0.7	0.19-2.82	0.667
Irregular	3	14	Ref		
**Treatment of drinking water**					
Yes	35	101	1.9	0.98-3.78	0.058
No	135	693	Ref		
**How many people**					
≤5(242)	36		Ref		
6-7(109)			2.1	0.94-4.94	0.068
8-10 (171)			2.3	1.10-4.92	0.026
>10 (229)			2.5	1.18-5.35	0.017

## Discussion

The result from our study revealed that most of the surveyed households had low socioeconomic status, with more than half of them earning below the minimum wage and farming being the main source of income. We found that the overall prevalence of diarrheal disease in the two weeks prior to the survey at household level was 17.3%. This prevalence is similar to what was found in a study conducted in South Africa, where the prevalence was 20% [12]. The prevalence of self-reported diarrheal disease in our study was lower than a study conducted in Ethiopia (33%) [13]. In our study, the prevalence in rural settings (20.5%) was higher. This pattern was seen across all age groups and was similar to the findings of a study conducted in India [14].

According to our study, the prevalence rate is the under-five age group (14.9%), which is similar to the situation in Indonesia (14.4%) [15]. Our findings are higher than those observed in Nigeria, where the prevalence of diarrhea in under-five age group is 11.3% [6]. Our findings are different from those observed in a study conducted in Nepal, in which the 15-44 age group was most affected. Our findings may be different because the Nepal study was conducted within the context of a suspected outbreak investigation [16]. This increase in the diarrheal diseases among under five children might be due to transition from breastfeeding introduction of new types of food such as complementary and solid food which is unsafe or in poor hygiene. Mothers who do not to consider children hygiene, such as washing hand after defecating, increase the risk of diarrheal diseases.

Our study indicated that 80% of the households surveyed had clean drinking water containers, and that the majority of them (82.8%) did not treat water before drinking. According to results, there are very few water treatment practices at the point of use. What´s more, water storage practices may have compromised the quality of water sourced from improved water sources. Our findings are similar to a study conducted in India, in which 74.2% of households reported cleaning water storage appliances at least once a day, and 69% households reported that no measures were taken at home to ensure drinking water safety [17]. Contamination may occur when water is put into the container or taken out of the container, thereby reducing the protective effects of the treatment [18].

Shared sanitation facilities are associated with an increase in diarrheal diseases, but this association was not statistically significant [19]. Approximately, 70% of households had a shared facility in Sierra Leone [20]. Our findings are greater than what was found in a study conducted in India where 25% of the study respondents shared toilets [17]. Shared toilets create an unhygienic environment for pathogen related to diarrheal diseases, which increase the possibility of pathogens being transmitted from infected persons to others. It was shown by other studies that households with a low socio-economic class were more likely to use unimproved sanitation, which means that the level of wealth can influence access to improved sanitation [21]. Our study shows that the majority (93.1%) of respondents wash their hands with water and soap, and nearly one-third (30.2%) of the respondents wash their hands with water only. Studies show that the prevalence of handwashing with soap in low and middle-income countries is between 13% and 17% [22].

Although an association has been observed between households treating water and diarrheal disease, this association is not statistically significant. Water contamination can occur during the process of treatment or during storage after treatment [23]. Our findings are inconsistent with the results of a study conducted in Ecuador, in which water treatment at households have a protective effect [24]. Furthermore, a systematic review observed that the risk of diarrheal disease was reduced in households where water treatment was performed [25].

Our study found that diarrheal disease is significantly associated with households have more than five people. This is inconsistent with results of studies conducted in Ethiopia and Nepal, in which family size was not associated with diarrheal disease [26]. The association seen in this study may have arisen as a result of compromised cleanliness and accessibility when a large number of people share sanitation facilities. The large family size causes family members to share toilets, providing a good environment for the spread of pathogens including diarrheal diseases. In our study, unimproved water source was associated with diarrheal disease. This is inconsistent with findings from studies in Burundi where they didn´t find any association between unimproved water sources and diarrheal disease [27]. Another study conducted in Ethiopia also found similar findings that families that use improved water sources are less likely to develop diarrheal diseases [28]. Low quality or unimproved drinking water provides environment for infectious pathogens to grow and circulate which can be a source of contamination for people drinking this water. Therefore, people who drink unimproved water will be exposed to pathogens that cause diarrhea.

The limitations of this study include that we cannot establish a causal association dependent and independent variables a cross-sectional study. The occurrence of the diarrheal diseases in this study was based on self-reports of diarrhea in the last two weeks before the investigation, which may cause recall bias. In addition, the occurrence of diarrheal disease may vary from season to season, which will affect the results of the study findings and affect the generalizability of the study.

## Conclusion

This study determined the prevalence of diarrheal diseases in Sierra Leone, which is higher than the average in sub-Saharan Africa. We concluded that the main risk factors for diarrheal diseases included unimproved water, and large family size. In Sierra Leone, diarrheal diseases remain a serious health problem. This requires enhanced WASH intervention, particularly in rural areas. We recommend the Ministry of Health and Sanitation and other Governmental partners to increase the access to improved water sources, improved sanitation, and hygiene.

### What is known about this topic

- Improved water resources, sanitation and hygiene have a role reducing diarrheal disease;
- Diarrheal disease considered a major health problem in Sierra Leone.

### What this study adds

- Diarrheal disease is associated with unimproved water and family size in Sierra Leone;
- Prevalence of diarrheal was high in children less than five years;
- Rural areas have more diarrheal diseases compared to urban area.
